# Exploration of the Reduction Diffusion Temperature for Different Phases of Samarium–Cobalt Magnetic Particles

**DOI:** 10.3390/molecules30091975

**Published:** 2025-04-29

**Authors:** Yani Lu, Xiangyu Ma, Jinping Ren, Jinke Kang, Yatao Wang

**Affiliations:** 1 Gansu Key Laboratory of Efficient Utilization of Oil and Gas Resources, College of Petroleum and Chemical Engineering, Longdong University, Qingyang 745000, China; 2 Shanxi Aerospace Qinghua Equipment Co., Ltd., Changzhi 046000, China; maxiangyu7843@foxmail.com; 3 Key Laboratory of Advanced Functional Materials, Ministry of Education of China, College of Materials Science and Engineering, Beijing University of Technology, Beijing 100124, China

**Keywords:** microwave-assisted combustion, samarium–cobalt particles, coercivity, rare earth materials

## Abstract

We report a method for synthesizing different phases of samarium–cobalt particles through microwave-assisted combustion combined with high-temperature reduction and diffusion, and identify the optimal temperature for forming the 1:5 phase using this approach. Initially, the samarium-to-cobalt ratio in a nitrate solution was determined. Using urea as both a reductant and fuel, samarium–cobalt oxides were synthesized via microwave-assisted combustion. The main components of the oxides were confirmed to be SmCoO_3_ and Co_3_O_4_. Subsequently, samarium–cobalt particles were synthesized at various diffusion temperatures. The results indicate that at 700 °C, the oxides were reduced to elemental Sm and Co. As the reduction temperature increased, the alloying of samarium and cobalt occurred, and the particle size gradually increased. At 900 °C, a pure 1:5 phase was formed, with particle sizes of approximately 800 nm, a coercivity of 35 kOe, and a maximum energy product of 14 MGOe. Based on the microwave-assisted combustion method, this study clarifies the transition temperatures of samarium–cobalt phases during the reduction and diffusion process, and further establishes the synthesis temperature for the 1:5 phase, providing new insights into the preparation and development of samarium–cobalt materials and potentially other rare earth materials.

## 1. Introduction

Samarium–cobalt (SmCo)-based compounds have long been recognized as one of the premier hard magnetic materials, owing to their exceptionally high magnetocrystalline anisotropy and elevated Curie temperatures [1,2,3]. These unique properties make SmCo-based magnets highly suitable for applications where strong and stable magnetic fields are required, such as in motors, generators, and various high-performance magnetic devices.

The magnetic properties of SmCo alloys can be further enhanced through the precise control of their microstructure at the nanometer level, and by synergizing the nanomagnets with a soft iron-based phase [4,5,6,7,8]. This fine-tuning of the microstructure allows for the optimization of the magnetic performance, tailored to specific application needs.

In recent years, considerable research efforts have been directed towards the synthesis of nanostructured Sm-Co materials, aiming to exploit their enhanced magnetic properties. One promising approach involves the combination of solvothermochemical synthesis with a high-temperature reduction process [9,10,11,12,13]. Microwave-assisted combustion synthesis (MACS), an innovative heating technology of significant importance, has evolved from self-propagating high-temperature synthesis (SHS) and low-temperature combustion synthesis (LTCS). This technique is characterized by its ability to rapidly elevate the reactant temperature to the ignition point under microwave irradiation, triggering spontaneous combustion within milliseconds to initiate the reaction and produce the desired products. The primary reactants—nitrate salts and organic fuels—are dissolved in a minimal volume of deionized water to form a slurry. Upon microwave heating, this slurry undergoes rapid decomposition, generating substantial combustible gases accompanied by exothermic heat release. Once the auto-ignition temperature is reached, the reactants combust spontaneously, completing the entire reaction process within minutes. In this system, nitrate salts function as oxidants, while glycine, urea, citric acid, and glycine acid serve as reducing agents. This method not only allows for the production of nanostructured Sm-Co particles, but also offers the potential for large-scale synthesis, making it attractive for industrial applications.

However, despite the significant progress made in the synthesis of nanostructured Sm-Co materials, there remains a need for a deeper understanding of the factors influencing their magnetic properties. One critical parameter that has garnered considerable attention is the reduction diffusion temperature. The reduction diffusion temperature plays a pivotal role in determining the phase composition, microstructure, and ultimately, the magnetic properties of the resulting Sm-Co particles.

In this study, we aim to explore the influence of the reduction diffusion temperature on the different phases of samarium–cobalt magnetic particles. By systematically varying the reduction diffusion temperature, we seek to gain insights into the phase transformation mechanisms, microstructural evolution, and magnetic property enhancements associated with this parameter. It was found that at 700 °C, the oxide decomposed into elemental samarium and cobalt. As the temperature further increased, the alloying of samarium and cobalt occurred. When the temperature reached 900 °C, pure SmCo_5_ was synthesized, exhibiting a coercivity of 35 kOe and a maximum energy product of 14.2 MGOe. With a further increase in temperature, a mixed phase of SmCo_5_ and Sm_2_Co_17_ was obtained.

Our findings will not only contribute to the fundamental understanding of Sm-Co magnet synthesis, but also provide practical guidelines for the optimization of their magnetic properties, paving the way for the development of advanced magnetic materials with superior performance.

## 2. Results and Discussion

**Figure 1:**
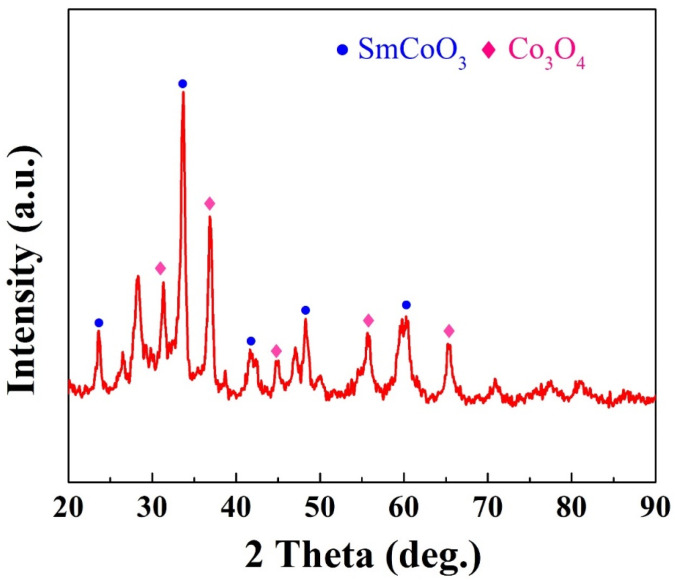
XRD pattern of as-prepared SmCo-O particles.

The combustion reaction yielded SmCoO_3_ and Co_3_O_4_, with the generated gas being a mixture of N_2_, CO_2_, and H_2_O. The synthesis of SmCo-O nanoparticles involves two key stages: nucleation and growth. Urea is crucial for controlling crystal formation and expansion by modifying the solution’s alkalinity, with hydroxide ions from urea hydrolysis playing a vital role in nucleation. SmCo-O nucleates from samarium cobalt basic ion solutions into polynuclear aggregates. Microwave radiation accelerates the production of polycrystalline SmCo-O nanostructures, converting reactants into loose nanocrystalline particles within minutes. Combustion using a flame also enhances the formation rate [14,15,16,17]. The flaky appearance of SmCo-O is attributed to gas release during the reaction. The XRD pattern shows that the product mainly consists of SmCoO_3_ and Co_3_O_4_ phases, as indicated in Figure 1. The content ratio of the two oxides is close to 1:1 [11]. Other diffraction peaks with relatively lower intensities correspond to the small amount of Sm_2_O_3_ and CoO phases [11,14].

**Figure 2:**
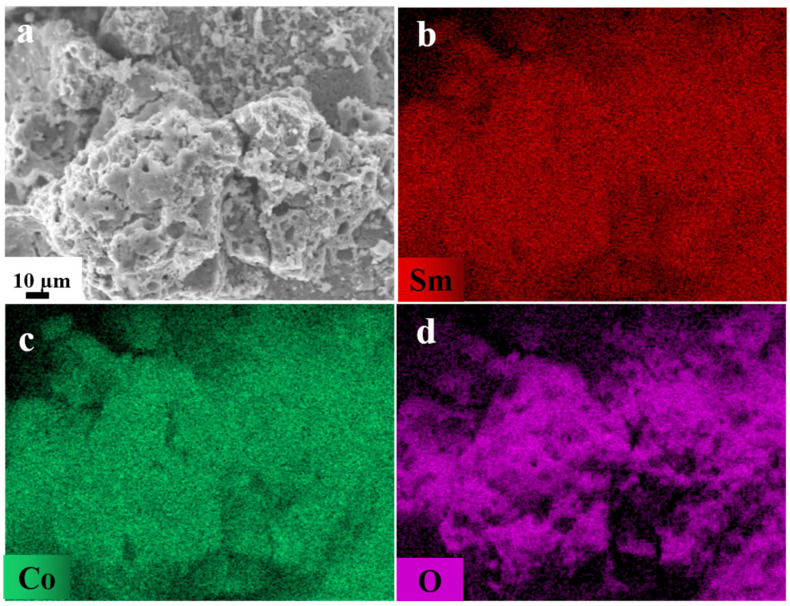
(**a**) SEM image and elemental mapping of (**b**) Sm, (**c**) Co, and (**d**) O correspond to the SEM image shown in (**a**).

The SmCo-O particles were subjected to further analysis using scanning electron microscopy (SEM). An SEM image of the particles, revealing a loose pore structure, is presented in Figure 2a. This structure is attributed to the emission of a significant quantity of gases during the combustion reaction. Energy dispersive spectroscopy (EDS), integrated with SEM, was utilized to examine the elemental composition of the precursor oxides. As depicted in Figure 2b–d, the elements Sm, Co, and O exhibit a homogeneous distribution. The generation of gases inhibited particle growth and aggregation, thus yielding loose precursor oxides.

**Figure 3:**
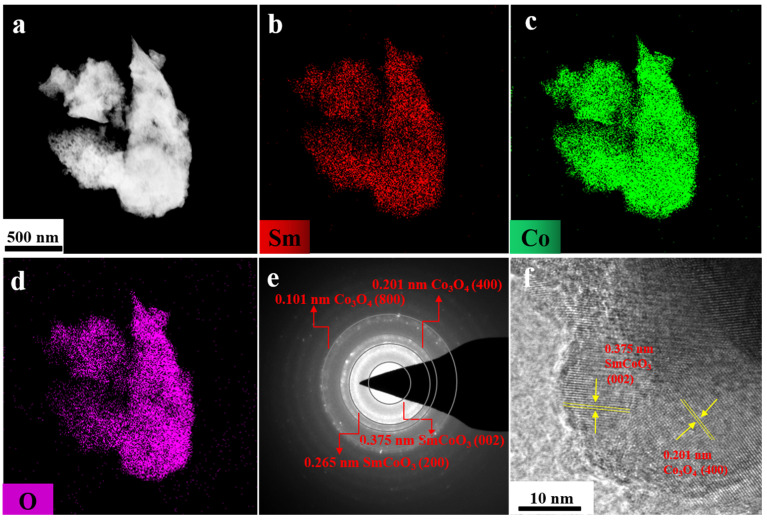
Characterization of as-prepared Sm-Co oxide precursor: (**a**) HAADF-STEM image and elemental mapping of (**b**) Sm, (**c**) Co, (**d**) O, (**e**) SAED pattern of the Sm-Co oxide particles, and (**f**) HRTEM image of a part of the representative particle.

To investigate the phase composition of samarium–cobalt oxide powder, a transmission electron microscopy (TEM) experiment was conducted, and the results are presented in Figure 3. The high-angle annular dark-field scanning transmission electron microscopy (HAADF-STEM) image (Figure 3a) and elemental mapping demonstrate the homogeneous distribution of samarium (Sm) (Figure 3b), cobalt (Co) (Figure 3c), and oxygen (O) (Figure 3d). It is noteworthy that the samarium–cobalt oxide precursor synthesized via microwave-assisted combustion exhibits a uniform distribution of Sm and Co, along with separated SmCoO_3_ and Co_3_O_4_ phases, which is crucial for facilitating interdiffusion between Sm and Co during the subsequent reduction step and the formation of the magnetic SmCo phase [10,18,19,20,21,22]. The nanostructure of the oxide powder may be attributed to the generation of gaseous by-products, such as carbon dioxide, nitrogen, and water vapor, during the microwave-assisted combustion process, which can hinder particle growth and aggregation, thereby favoring the formation of small-sized, nanoscale oxide powder [23]. Selected area electron diffraction (SAED) indicates that the oxide predominantly comprises Co_3_O_4_ and SmCoO_3_ phases, as shown in Figure 3e. Furthermore, high-resolution transmission electron microscopy (HRTEM) images provide additional confirmation that the oxide particles contain mixed grains of Co_3_O_4_ and SmCoO_3_ (Figure 3f), consistent with the previous XRD analysis results.

**Figure 4:**
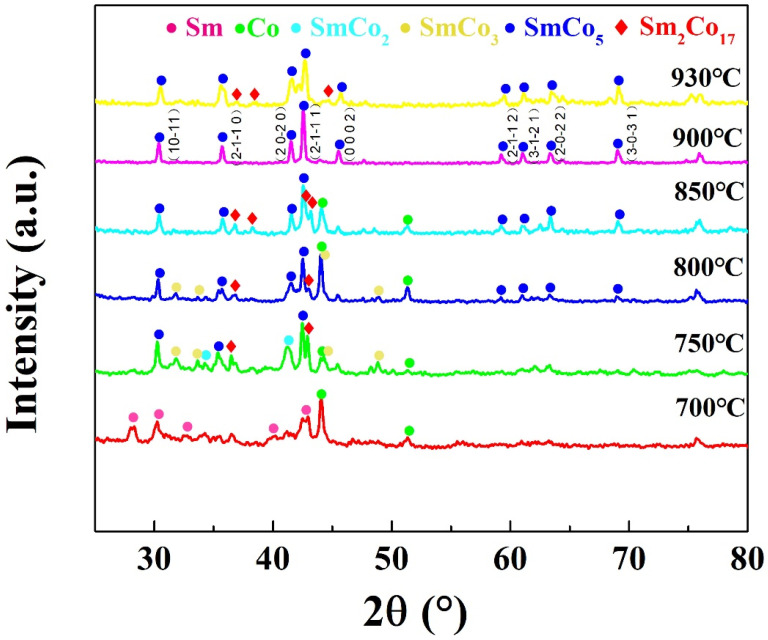
The XRD patterns of SmCo particles synthesized at different temperatures.

**Figure 5:**
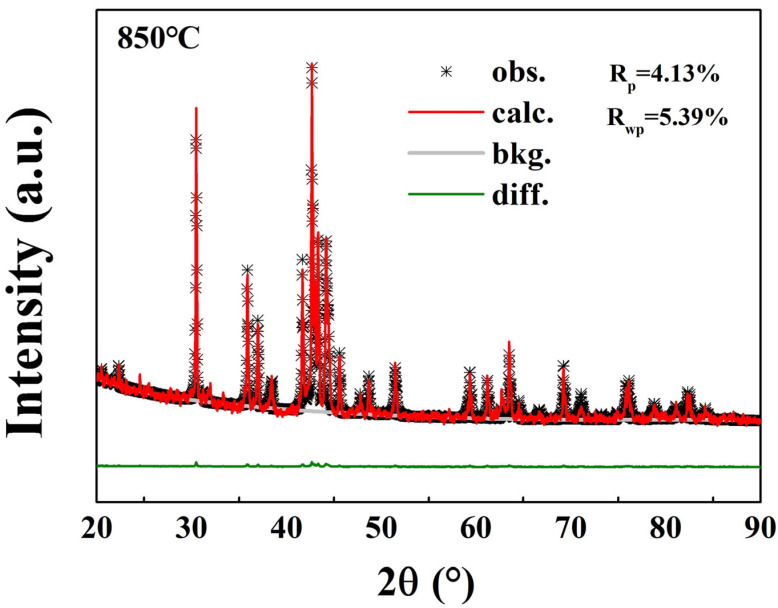
Rietveld analysis of the XRD pattern of SmCo particles synthesized at 850 °C. The black *, red line, and green line correspond to the experimental data, the calculated line, and the difference between the fitted and experimental results, respectively. The goodness-of-fit of R_p_ and R_wp_ is illustrated.

**Figure 6:**
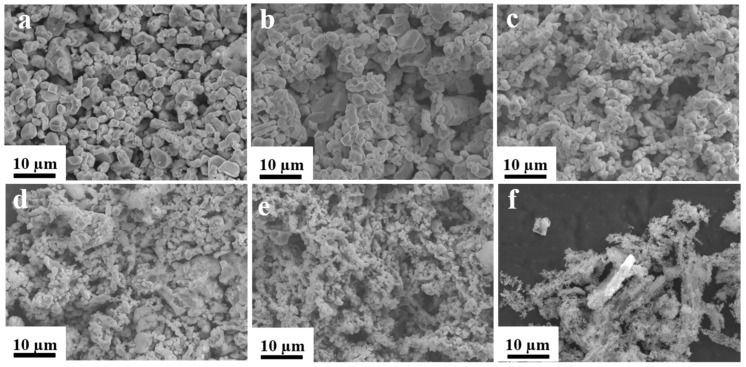
The SEM images of SmCo particles synthesized at different temperatures. ((**a**) 930 °C, (**b**) 900 °C, (**c**) 850 °C, (**d**) 800 °C, (**e**) 750 °C, and (**f**) 700 °C).

Samarium–cobalt oxide was reduced at different temperatures and held for 90 min. Water and ethanol were used to remove the residual reducing agents and non-magnetic impurities, respectively. To ascertain the structure and morphology of the products, XRD (as shown in Figure 4 and Figure 5) and SEM tests (as shown in Figure 6) were conducted. The results indicated that when the reduction temperature was 700 °C, the morphology consisted of irregular, slender, and amorphous particles, with the phases being metallic elemental samarium and cobalt, and no oxide phase present, suggesting that the samarium–cobalt oxide had been completely reduced at this point. As the temperature increased, the particles of the product gradually took shape and grew larger, and the samarium and cobalt elements gradually alloyed, resulting in multiple alloy phases. Between 700 °C and 800 °C, alloy phases of samarium–cobalt begin to appear, but the SmCo_5_ phase is not distinctly evident, indicating that within this temperature range, the SmCo_5_ phase cannot exist stably, which is consistent with reports in the literature [24,25]. In order to accurately investigate the effect of temperature on the phase composition and phase content, Rietveld refinement was conducted on the magnetic powder obtained at 850 °C, as shown in Figure 5. Here, “*” denotes the measured diffraction peak data, the red line represents the computer-fitted data obtained via a point-by-point comparison of diffraction peak intensities using X’Pert Highsore Plus 4.9 software computer software, and the green line indicates the error between the measured data and the calculated/fitted data. The refined structure contains the SmCo_5_, Co, and Sm_2_Co_17_ phases, with their respective contents being 75.6%, 15.7%, and 8.7%. When the temperature rose to 900 °C, the particles became more uniform, with a size distribution ranging from 500 nm to 1000 nm, forming a pure 1:5 phase. The diffraction pattern exhibited by the prepared product can be accurately indexed in accordance with the single-phase structure of SmCo_5_ (space group P6/mmm, No. 191). Continuing to elevate the reduction temperature led to an increase in particle size and the formation of a 2:17 phase in addition to the 1:5 phase, attributed to the volatilization of samarium at high temperatures [24,25].

**Figure 7:**
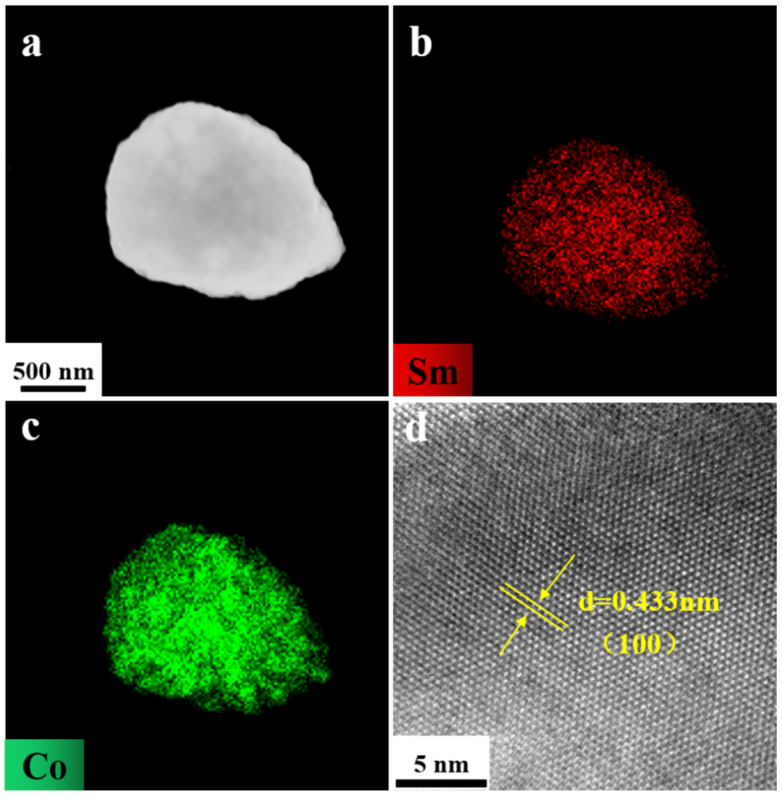
Characterization of as-prepared SmCo_5_: (**a**) HAADF-STEM image and elemental mapping of (**b**) Sm, (**c**) Co, and (**d**) HRTEM image of a part of the representative particle.

As illustrated in Figure 4, the X-ray diffraction (XRD) pattern of the as-prepared product exhibits a well-defined set of peaks that can be readily indexed to the single phase of SmCo_5_ (space group P6/mmm, No. 191). This confirms the successful synthesis of the targeted compound with high phase purity. The scanning electron microscopy (SEM) image presented in Figure 6b provides a closer look at the morphology of the SmCo_5_ particles. It is evident that the product adopts an ellipsoid shape with a uniform particle size of approximately 800 nm, which is notably just below the threshold for single-domain behavior. To further investigate the elemental distribution within the SmCo_5_ particles, high-angle annular dark-field scanning transmission electron microscopy (HAADF-STEM) and elemental mapping were performed on an individual particle after ultrasonic dispersion. The results, depicted in Figure 7a–c, reveal a homogeneous distribution of both Sm and Co elements throughout each SmCo_5_ particle [26,27]. This uniform elemental distribution is crucial for achieving optimal magnetic properties and stability in the final product. Moreover, the high-resolution transmission electron microscopy (HRTEM) image shown in Figure 7d offers insights into the crystalline structure of the SmCo_5_ particles. The observed interplanar spacing of 0.433 nm corresponds to the (100) plane of the SmCo_5_ crystal structure, further confirming the phase identity and crystalline quality of the synthesized material [28,29,30,31,32].

**Figure 8:**
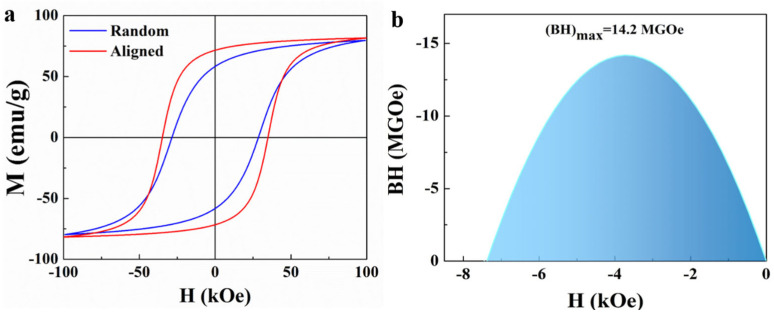
(**a**) Magnetic hysteresis loop of composed and aligned SmCo_5_ particles. (**b**) BH curve of the aligned SmCo_5_ particles.

The magnetic properties of the synthesized SmCo_5_ particles were rigorously evaluated using a physical property measuring system (PPMS) under an applied magnetic field of up to 100 kOe to gain a comprehensive understanding of their performance. The results obtained from this analysis are presented in Figure 8. As depicted in Figure 8a, the coercivity of the as-synthesized (unoriented) SmCo_5_ particles was measured to be 29 kOe, indicating their resistance to demagnetization and their potential for applications requiring high magnetic stability. Furthermore, to enhance the magnetic properties, the SmCo_5_ particles underwent an orientation process, which is known to improve the alignment of the magnetic domains within the particles, thereby enhancing their magnetic performance [9,33]. When nanoparticles are aligned by external fields (e.g., magnetic or mechanical force fields), their magnetic moments preferentially orient along specific crystallographic axes (i.e., easy magnetization axes). This ordered arrangement significantly elevates the magnetic anisotropy energy (Ku) by creating a higher energy barrier that must be overcome for magnetic moment reversal. The detailed processes are as follows: The SmCo_5_ particles were dried in a vacuum oven at 323 K. A certain mass of SmCo_5_ particles was dispersed in epoxy resin and then taken together into a cylindrical container. Subsequently, the container was placed in a static magnetic field of about 2.2 T, provided by a permanent magnet for 4 h. The cylindrical sample was removed from the magnetic field after the epoxy resin was fully solidified. Following this orientation treatment, the coercivity of the particles increased to 35 kOe for the SmCo_5_ phase, demonstrating the effectiveness of the orientation process in improving the magnetic characteristics [31,32,33]. In addition to the coercivity, the maximum magnetic energy product (BH)max is another critical parameter for evaluating the performance of magnetic materials. As illustrated in Figure 8b, the oriented SmCo_5_ particles exhibited a (BH)_max_ of 14.2 MGOe, which is a significant indicator of their potential for high-performance magnetic applications. These results not only highlight the excellent magnetic properties of the synthesized SmCo_5_ particles, but also underscore the importance of the orientation process in optimizing their magnetic performance for various applications.

The aligned SmCo_5_ particles exhibit a relatively high coercivity of 35 kOe. In comparison with other research findings, Table 1 summarizes the key factors for SmCo_5_ particles synthesized via chemical methods, including raw materials, forms of precursors, and coercivity [8,9,11,21,22,34]. Compared with other relevant studies [8,21,22,34], this work has the advantage in terms of raw material utilization, as it avoids the use of acids and bases. Moreover, it does not employ additional nitrate coating on calcium oxide, thereby simplifying the synthesis process and conserving resources [9,11]. These SmCo_5_ magnetic particles hold promising potential for high-performance permanent magnet applications and can be utilized in the development of exchange-coupled nanocomposite magnets with high energy density.

## 3. Materials and Experiment

### 3.1. Synthesis of SmCo-O

The microwave-assisted combustion synthesis (MACS) method was employed to fabricate SmCo-O nanoparticles using samarium nitrate hexahydrate (Sm(NO_3_)_2_·6H_2_O) and cobalt nitrate hexahydrate (Co(NO_3_)_2_·6H_2_O) as metallic precursors, with urea serving as a combustion promoter. Stoichiometric quantities of 2.0 g of Sm(NO_3_)_2_·6H_2_O, 4.19 g of Co(NO_3_)_2_·6H_2_O, and 2.28 g of urea were dissolved in 2 mL deionized water within a porcelain crucible to form a viscous pasty precursor. This mixture was subjected to microwave irradiation at 700 W, triggering exothermic self-sustaining reactions that completed combustion within 2 min and produced black, loose SmCo-O nanoparticles as residues. The nanoparticles were initially dispersed in 400 mL of absolute ethanol and homogenized via mechanical stirring for 60 min. Subsequent centrifugation at 3000 rpm for 3 min was conducted, followed by the ambient-temperature drying of the oxide powder, which was then reserved for subsequent experiments.

### 3.2. Synthesis of SmCo

The as-synthesized SmCo-O particles were blended with KCl and Ca to form a composite mixture. This mixture was transferred into an iron crucible equipped with a hermetic cover, which was subsequently loaded into a tube furnace for thermal processing. Prior to heating, the furnace tube was evacuated to achieve a vacuum state, followed by purging with an Ar/H_2_ gas mixture to establish a protective atmosphere. The furnace was then heated to the target temperature at a ramp rate of 8 K min^−1^, maintained at this temperature for 1.5 h to ensure a complete reaction, and allowed to cool naturally to 25 °C. The post-treatment procedure comprised the centrifugation-based removal of non-magnetic impurities (parameters: 8000 rpm for 5 min), culminating in the acquisition of the final SmCo magnetic powder that was subsequently stored in anhydrous ethanol.

### 3.3. Characterization

X-ray diffraction (XRD) analysis was performed using a Rigaku Ultima IV instrument (Akishima, Japan) to investigate the structural characteristics of oxide and alloy particles. Scanning electron microscopy (SEM, model ZEISS-SUPRA55, Jena, Germany) and transmission electron microscopy (TEM, model Tecnai-F20, FEI, Hillsboro, OR, USA) were employed to observe the morphology and analyze the microstructure of the samples. A certain mass of SmCo_5_ particles were uniformly dispersed in epoxy resin and subsequently placed into a cylindrical container. The container was then subjected to a static magnetic field of 2.2 T. After 4 h, the epoxy resin was fully cured, and the sample was removed and cut into small rectangular prisms. The magnetic properties of these prisms were measured using a physical property measurement system (PPMS, manufactured by Quantum Design, San Diego, CA, USA).

## 4. Conclusions

In this study, we have successfully demonstrated a method for synthesizing different phases of samarium–cobalt particles through the combination of microwave-assisted combustion and high-temperature reduction and diffusion. By initially determining the samarium-to-cobalt ratio in a nitrate solution and using urea as both a reductant and fuel, we were able to synthesize samarium–cobalt oxides via microwave-assisted combustion, with the main components confirmed to be SmCoO_3_ and Co_3_O_4_. Subsequent synthesis at various diffusion temperatures revealed that at 700 °C, the oxides were reduced to elemental Sm and Co, and that as the reduction temperature increased, the alloying of samarium and cobalt occurred with a gradual increase in particle size. Notably, at 900 °C, a pure 1:5 phase was formed, exhibiting particle sizes of approximately 800 nm, a coercivity of 35 kOe, and a maximum energy product of 14.2 MGOe. This study not only clarifies the transition temperatures of samarium–cobalt phases during the reduction and diffusion process based on the microwave-assisted combustion method, but also establishes the synthesis temperature for the 1:5 phase. These findings provide new insights and approaches for the preparation and development of samarium–cobalt materials and hold potential implications for the synthesis of other rare earth materials.

## Figures and Tables

**Table 1:** The raw materials, precursors, and coercivity for SmCo_5_ particles synthesized via chemical methods.

Starting Raw Materials	Precursors	Coercivity (kOe)	Ref.
Sm(acac)_3_·xH_2_O, Co(acac)_2_, GO, Ca, KCl, oleylamine, oleic acid	Sm_2_O_3_-Co@GO	24.4	[21]
Sm(NO_3_)_3_·6H_2_O, CoCl_2_·6H_2_O, NaOH, KCl, Ca	Sm(OH)_3_, Co(OH)_2_	33.2	[8]
Co(ac)_2_, Sm(ac)_3_, n-hexadecyltrimethylammonium hydroxide, 1-octadecene, oleic acid, KCl, Ca	SmCo–O	7.2	[22]
Sm(NO_3_)_3_·6H_2_O, Co(NO_3_)_2_·6H_2_O, PVP,CoCl_2_ C_2_H_5_OH, CO(NH_2_)_2_,NaOH,C_11_H_23_COOH,Ca	SmCoO_3_, Co_3_O_4_, Sm_2_O_3_, Co	34.5/47.2	[9]
Sm(NO_3_)_3_, Co(NO_3_)_2_, citric acid, Ca, CaO	SmCoO_3_, Co_3_O_4_	29	[34]
Sm(NO_3_)_3_·6H_2_O, Co(NO_3_)_2_·6H_2_O, CO(NH_2_)_2_, Ca(NO_3_)_2_, CaH_2_	SmCoO_3_, Co_3_O_4_	39.2	[11]
Sm(NO_3_)_3_·6H_2_O, Co(NO_3_)_2_·6H_2_O, CO(NH_2_)_2_, Ca	SmCoO_3_, Co_3_O_4_	35.0	This work

## Data Availability

Data is contained within the article.
